# Study of Function and Regulatory Factors of *CaPEX3* in the Regulation of Pollen Viability in Pepper (*Capsicum annuum* L.)

**DOI:** 10.3390/plants14223441

**Published:** 2025-11-10

**Authors:** Qiao-Lu Zang, Lu Liu, Meng Wang, Xiao-Mei Zheng

**Affiliations:** College of Horticulture, Shanxi Agricultural University, Taigu, Jinzhong 030801, China; l13593562360@163.com (L.L.); wm102300@163.com (M.W.); zxm2809968194@163.com (X.-M.Z.)

**Keywords:** pepper, *CaPEX3*, pollen viability, transcription factor, breeding

## Abstract

The vitality of pollen significantly influences the efficiency of pollination and microspore embryogenesis. Mining genes associated with pollen vitality will help accelerate pepper (*Capsicum annuum* L.) breeding progress via genetic engineering. *PEX* (*pollen extensin-like*), a member of the LRX (leucine-rich repeat extensin) family, is predominantly expressed in pollen and participates in regulating pollen vitality. However, its function and regulatory factors in pepper remain elusive. In this study, GUS histochemical staining results revealed that pepper *CaPEX3* could be expressed in petals, sepals, anthers, and pollens of transgenic tomato (*Solanum lycopersicum* L.) lines expressing *CaPEX3 promoter*::*GUS*. Moreover, inhibition of the *CaPEX3* by virus-induced gene silencing (VIGS) in pepper resulted in reduced pollen germination rate and viability, while overexpression of *CaPEX3* in tomato significantly enhanced germination rate and pollen viability. In addition, *TRANSPARENT TESTA GLABRA 1* (*CaTTG1*) and *Nuclear transcription factor Y subunit C9* (*CaNFYC9*) were screened out and identified as the upstream regulatory transcription factors of *CaPEX3* through yeast one-hybrid (Y1H) screening and dual-luciferase reporter (Dual-LUC) assays. Taken together, the identification of transcription factors may reveal a more comprehensive mechanism underlying *CaPEX3*-mediated enhancement of pepper pollen viability. This study not only provides genetic resources for pollen viability research but also establishes a theoretical foundation for pepper breeding.

## 1. Introduction

Pepper (*Capsicum annuum* L.), as one of the world’s most extensively cultivated vegetable crops, is highly valued by consumers for its nutritional richness and processing versatility [1]. Currently, pepper breeding primarily relies on hybrid pollination, which is labor-intensive and time-consuming, with efficiency constrained by factors such as pollen viability [2,3]. Haploid breeding offers a solution to shorten breeding cycles and accelerate progress [4,5]. Although microspore embryogenesis serves as a key method for haploid production, its application is severely limited by low embryo induction rates [6,7]. Crucially, microspore embryogenesis efficiency correlates with pollen developmental stage and vitality [8,9]. Thus, it is of vital importance to study the influencing factors of pollen vitality.

Pollen development is a complex process that can be generally divided into three stages: (i) microsporogenesis; (ii) post-meiotic development of microspores; and (iii) microspore mitosis; and these stages require a great deal of resources such as sugars, lipids, and proteins [10,11]. A viable pollen signifies its capacity to successfully complete the entire complex process, from being released from the anther, germinating on the stigma, and growing a pollen tube to, ultimately, delivering the sperm cells to the ovule for double fertilization [11,12]. Pollen viability is coregulated by both external environmental factors and the plant’s intrinsic genetic background [13,14]. Environmental stresses such as high temperature can disrupt the cellular structure of pollen grains, interfere with energy metabolism, and lead to the accumulation of reactive oxygen species (ROS), thereby causing membrane lipid peroxidation and the loss of pollen viability [13,15]. Furthermore, the homeostasis of metabolites (such as flavonoids and anthocyanins) within the pollen is fundamental to sustaining its viability by modulating processes, including ROS levels [16,17,18]. Additionally, the essence of pollen development lies in the ordered spatiotemporal expression of specific genes, a process primarily orchestrated by transcription factors (TFs) and dependent on a sophisticated molecular regulatory network [19,20,21]. The Dysfunctional tapetum 1 (DYT1)-Tapetal development and function 1 (TDF1)-aborted microspores (AMS) -male sterile (MS) 188-MS1 cascade is a prototypical transcriptional hierarchy that sequentially controls tapetum specification, sporopollenin production, and pollen exine formation [20,22,23]. Therefore, identifying the genes and their regulatory factors that regulate pollen viability is beneficial for enhancing pollen vitality through genetic engineering, thereby improving pollination efficiency and microspore production and, ultimately, accelerating the breeding process.

Leucine-rich repeat extensins (LRXs) are proteins that contain a leucine-rich repeat domain and an extensin domain, and the involvement of LRXs in plant development, pathogen defense, and signal transduction has been reported [24,25]. Members of the LRX gene family were categorized into two major classes based on tissue specificity: those expressed in vegetative organs were denoted as LRX, while those in reproductive organs were termed PEX (pollen extensin-like) [26,27]. In *Arabidopsis thaliana*, *LRX8-11* (*PEX1-4*) were expressed in pollen and pollen tubes and critically regulated pollen germination and tube elongation [28]. In the triple mutants *lrx8 lrx9 lrx11* and *lrx9 lrx10 lrx11*, there was an increase in the amount of rhamnogalacturonan I in the subapical walls of pollen tubes; moreover, in the *lrx8 lrx9 lrx11* mutant, the content of callose increased, while that of fucosylated xyloglucans decreased, suggesting that *LRX8-11* function synergistically to maintain pollen tube cell wall integrity [28]. Furthermore, double mutant lrx8 lrx10, lrx9 lrx11, and lrx9 lrx10 and triple mutant *lrx9 lrx10 lrx11* showed a statistically significant reduction in pollen germination and an increase in abnormal pollen tubes [29]. Thus, mutations in the *LRX8-11* genes caused severe defects in pollen germination and pollen tube growth, resulting in a reduced seed set [30]. In rice (*Oryza sativa*), *OsPEX1* was highly expressed in pollen, and suppressing its expression caused pollen abortion and markedly reduced seed-setting rate [31]. Taken together, these results indicate that *PEX* may affect pollen viability, but its role in pepper remains unclear.

Notably, the interacting proteins of PEX in *A. thaliana* were reported. Rapid alkalinization factor 4/19 (RALF4/19) peptides interacted with LRX proteins to control pollen tube growth and cell wall integrity [32,33]. LRX8–RALF4–pectin interaction exerted a condensing effect, patterning the cell wall’s polymers into a reticulated network essential for wall integrity and expansion [34]. Moreover, LRXs were able to interact with the receptor-like kinase FERONIA to jointly sense and convey extracellular signals to the cell and then regulate cellular elongation [35,36]. In pear (*Pyrus bretschneideri*), ABRE binding factor PbABF.D.2. was able to directly bind the *PbLRXA2.1* and *PbLRXA2.2* promoters to stimulate pollen tube growth [37]. These results indicated that PEX may interact with other proteins and be regulated by TF to affect pollen development. However, whether the activity of *PEX* is regulated by transcription factors in pepper remains an intriguing question worthy of further investigation.

In the previous research, the *CaPEX3* gene was cloned from pepper, and it was found that the expression level of this gene in flowers was significantly higher than that in other tissues, and the pollen viability was found to be positively correlated with the expression level of *CaPEX3* [38]. In this study, the expression pattern of *CaPEX3* in flower-related tissues was analyzed. To further investigate the function of this gene, the expression of endogenous *CaPEX3* in pepper was silenced using the virus-induced gene silencing (VIGS) system. Furthermore, an overexpressing vector of *CaPEX3* was also constructed and transformed into tomato (*Solanum lycopersicum* L.). Then, the germination rate and pollen viability were analyzed in pepper lines with a lower expression of *CaPEX3* and in tomato lines with a higher expression of *CaPEX3* to determine the function of *CaPEX3* in regulating pollen viability. Additionally, a cDNA library of pepper was constructed to screen for the upstream regulatory transcription factors of *CaPEX3* using yeast one-hybrid (Y1H) assays. These results further clarified the function and mechanism of *CaPEX3*, providing genetic resources and establishing a theoretical foundation for pollen viability research in pepper.

## 2. Results

### 2.1. CaPEX3 Was Expressed in Flower-Related Tissues

**Figure 1:**
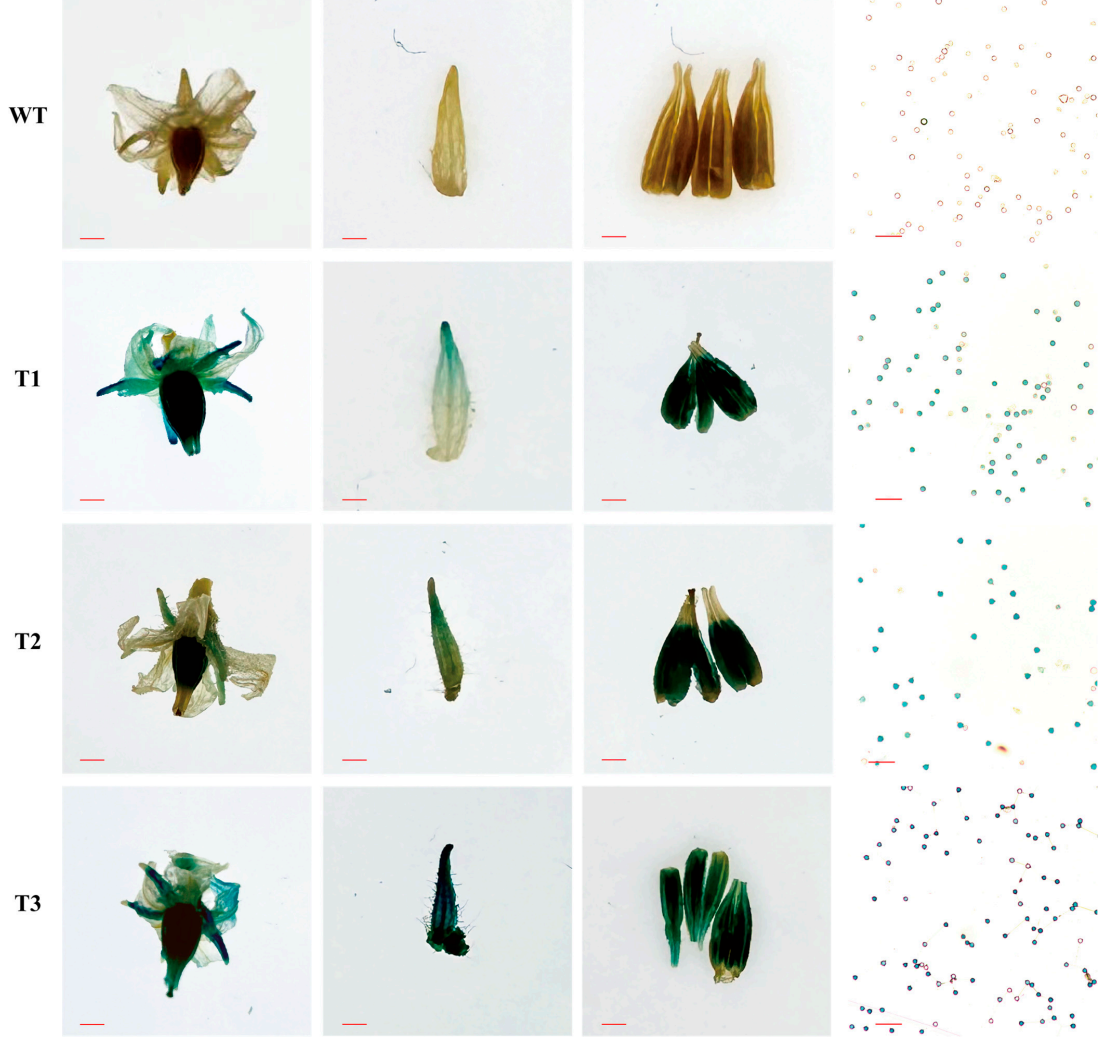
Activity analysis of *CaPEX3* promoter in tomato. GUS activity in flower-related tissues (flowers, sepals, anthers, and pollens; from left to right) of wild-type (WT) and three *CaPEX3-* overexpressing transgenic lines (T1–T3) of tomato. The red lines indicate scale bars: flowers, bar = 2 mm; sepals and anthers, bar = 1 mm; pollens, bar = 100 µm.

A previous study has revealed the predominant expression of *CaPEX3* in flowers [38]. To further determine its expression location in floral organs, a *CaPEX3 promoter*::GUS fusion construct was generated and transformed into tomato. The flower-related tissues of wild-type (WT) and transgenic lines (T1–T3) were stained, and the results showed that the petals, sepals, anthers, and pollens of the transgenic lines were all stained blue, whereas no staining was observed in the corresponding tissues of the WT (Figure 1). These results confirmed that the *CaPEX3* promoter exhibited promoter activity, capable of inducing *GUS* expression, and the gene was expressed in these test floral organs.

### 2.2. Inhibition of CaPEX3 Expression Reduced Pollen Vitality in Pepper

**Figure 2:**
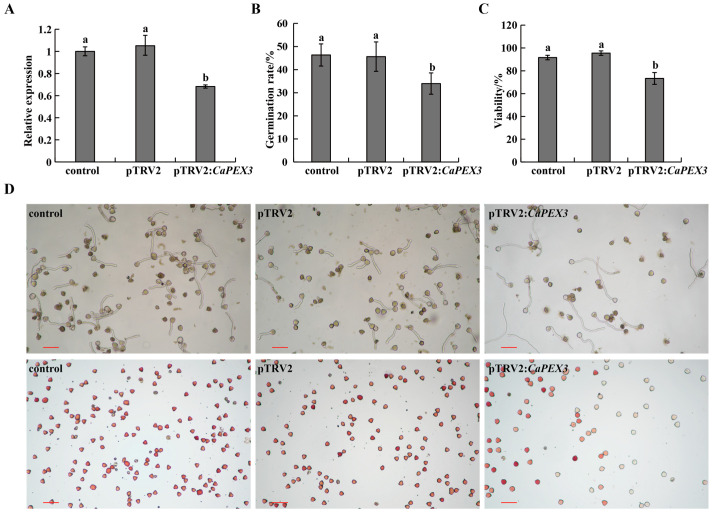
Effects of virus-induced gene silencing of *CaPEX3* on pollen viability in pepper. (**A**) qPCR analysis to detect the expression level of *CaPEX3* in flowers of the control /pTRV2/pTRV2:*CaPEX3* plants at 60 days post-infection. (**B**,**C**) Analysis of pollen germination rate (**B**) and pollen viability (**C**). (**A**–**C**): different lowercase letters indicated significant differences at *p* < 0.05 as determined by Duncan’s test. (**D**) Microscopic observation of pepper pollen germination and viability of the control (**left**), pTRV2 (**middle**), and pTRV2:*CaPEX3* (**right**) plants. The pollen viability was detected by 1% 2, 3, 5-triphenyltetrazolium chloride (TTC) staining method. Red, dark red pollen: viable pollens; uncolored pollens: non-viable pollens. The red lines indicate scale bars: bar = 100 µm.

In terms of pTRV2, the *CaPEX3* recombinant vector was designed to target the endogenous *CaPEX3* gene in pepper. According to the results of qPCR, at 60 days post-infection, the expression levels of *CaPEX3* in flowers were significantly reduced (by 31.72% in pTRV2:*CaPEX3*-infected plantlets) compared to the control (Figure 2A), demonstrating that *CaPEX3* can be effectively silenced using the VIGS system in pepper.

To further clarify the influence of *CaPEX3* on pollen vitality, pollen germination and vitality under different treatments were measured (Figure 2B–D). pTRV2:*CaPEX3*-infected plants exhibited a significantly reduced germination rate of 33.99%, significantly lower than that of both the control and pTRV2-infected groups (Figure 2B,D). Concurrently, pollen viability analysis using 1% 2, 3, 5-triphenyltetrazolium chloride (TTC) staining revealed that the rate of viable pollen of pTRV2:*CaPEX3*-infected plants was 73.34%, which was also significantly impaired compared to control groups (Figure 2C,D).

### 2.3. Overexpression of CaPEX3 Enhanced Pollen Viability in Tomato

**Figure 3:**
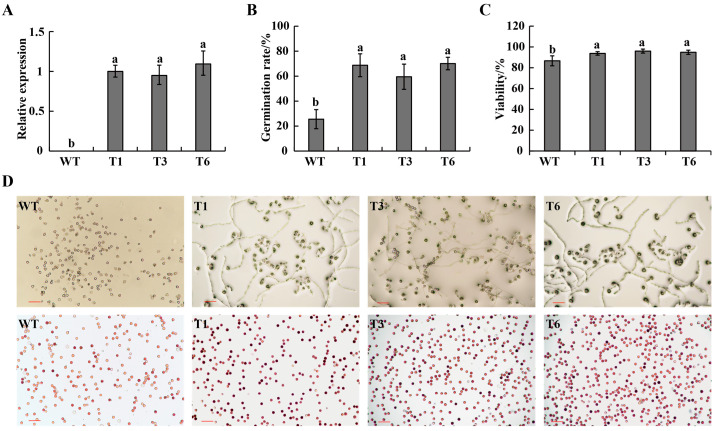
Effects of overexpression of *CaPEX3* on pollen viability in tomato. (**A**) qPCR analysis to detect the expression level of *CaPEX3* in flowers of WT and three *CaPEX3*-overexpressing transgenic lines (T1, T3, and T6). (**B**,**C**) Analysis of pollen germination rate (**B**) and pollen viability (**C**). A, B, and C: different lowercase letters indicated significant differences at *p* < 0.05 as determined by Duncan’s test. (**D**) Microscopic observation of tomato pollen germination and viability of WT and three *CaPEX3*-overexpressing transgenic lines (T1, T3, and T6). Pollen viability was detected by 1% 2, 3, 5-triphenyltetrazolium chloride (TTC) staining method. Red, dark red pollen: viable pollens; uncolored pollens: non-viable pollens. The red lines indicate scale bars: bar = 100 µm.

To further validate the function of *CaPEX3*, an overexpression vector of *CaPEX3* was constructed and transformed into tomato. As revealed in Figure 3A, the expression of *CaPEX3* was not detected in WT, while it was detected in the three *CaPEX3-* overexpressing transgenic lines (T1, T3, T6). The analysis of pollen germination in WT and transgenic lines (T1, T3, T6) revealed that the pollen germination rate of WT plants was 25.56%, while that of transgenic lines was 68.75%, 59.53%, and 70.12%, respectively, which were all significantly higher than that of WT (Figure 3B,D). Furthermore, pollen viability analysis revealed that the proportion of viable pollen of the wild type was 86.69%, while that of the transgenic lines was 93.76%, 96.00%, and 94.88%, respectively, higher than that of WT (Figure 3C,D).

### 2.4. Identification of Upstream Regulatory Transcription Factors of CaPEX3

To screen the upstream regulatory factors of *CaPEX3*, the *CaPEX3* promoter was used as a bait, and the Y1H assay was employed to conduct the screening in the pepper cDNA library. Finally, a total of 82 positive colonies were obtained and sequenced. The transcription factors among the sequences were predicted, and it was found that two sequences were annotated as transcription factors. Subsequently, these two sequences were cloned using pepper genomic information and annotated as *TRANSPARENT TESTA GLABRA 1* (*CaTTG1*) (GenBank accession number, PV577545) and *Nuclear transcription factor Y subunit C 9* (*CaNFYC9*) (GenBank accession number, PV577546). The region (220–1029 bp) of the full coding sequence (1029 bp) of *CaTTG1* and the region (1–596 bp) of the full coding sequence (777 bp) of *CaNFYC9* were shown to be able to bind the promoter of *CaPEX3*.

**Figure 4:**
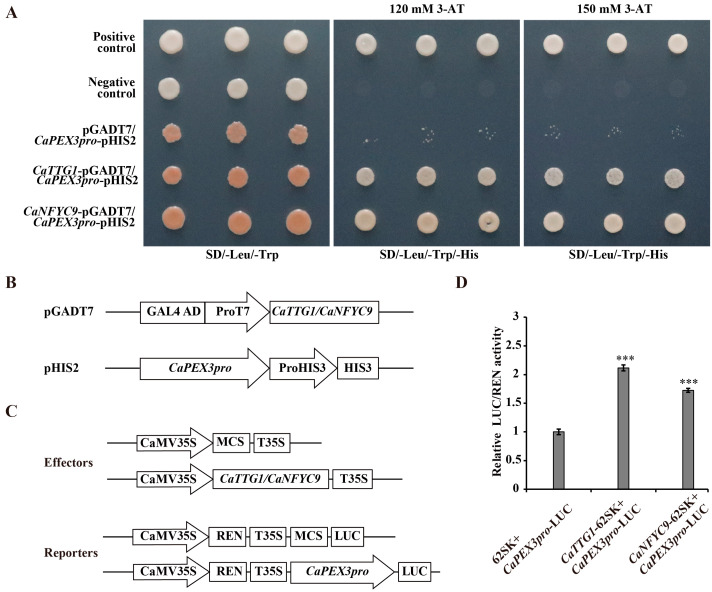
CaTTG1 and CaNFYC9 bind to the promoter of *CaPEX3* and activate its expression. (**A**) Yeast one-hybrid assays (Y1Hs) showed that CaTTG1 and CaNFYC9 bound to the promoter of *CaPEX3*. Rec2-53-pGADT7 and p53-pHIS2 were co-transformed into Y187 as positive control. pGADT7 and p53-pHIS2 were co-transformed into Y187 as negative control. (**B**) Schematic diagrams of the vectors used in Y1H assays. (**C**) Schematic diagrams of the effector and reporter vectors used in dual-luciferase reporter assays (Dual-LUC) assays. (**D**) Dual-LUC analysis was performed by means of transient infiltration of *Nicotiana benthamiana* leaves with equal concentrations of *Agrobacterium tumefaciens GV3101* cells transformed with effectors and reporters, respectively. The values were obtained as a ratio of the activity of firefly luciferase (LUC) and renilla luciferase (REN). Data represent values obtained as mean ± SD of three biological duplications. Error bars represented standard errors. *** *p* ≤ 0.001, Student’s *t*-test.

Furthermore, the relationship between transcription factors and *CaPEX3* was verified by using Y1H (Figure 4A,B). As illustrated in Figure 4A, no yeast growth was observed in the negative control or empty vector groups, whereas colonies were present in the positive control group. Additionally, yeast cells carrying the *CaTTG1*/*CaNFYC9* and *CaPEX3* promoter exhibited growth on the selective medium, suggesting that the CaTTG1 and CaNFYC9 proteins were both capable of binding to the *CaPEX3* promoter. To further validate these regulatory relationships, dual-luciferase reporter (Dual-LUC) assays were conducted, demonstrated that *CaTTG1*/*CaNFYC9* enhanced the promoter activity of *CaPEX3* (Figure 4C,D).

## 3. Discussion

Enhancing the vitality of pollen is of great significance for improving the efficiency of hybrid pollination and haploid breeding [3,5]. Mining genes that regulate pollen vitality in pepper will facilitate genetic engineering approaches to improve pollen vitality and breeding efficacy. *PEX* genes exhibited high expression in reproductive organs, particularly in pollen, and regulated pollen vitality [28,30]. In maize (*Zea mays*), the PEX1 protein is located inside pollen tubes and callose-rich pollen tube cell walls [39]. In *A. thaliana*, *AtLRX8-11* were highly expressed in pollen and pollen tubes, controlling pollen germination and tube elongation [28,30]. In addition, rice *OsPEX1* showed pollen-specific high expression [31]. Notably, the pepper *CaPEX3* gene exhibited high expression in flowers [38]. In this study, GUS histochemical staining confirmed that *CaPEX3* was expressed in flower-related tissues such as petals, sepals, anthers, and pollen. The genetic transformation of pepper should be employed to further validate the function of *CaPEX3*. However, current limitations in pepper transformation efficiency, which remains relatively low and technically challenging, had hindered its widespread application [40]. As a result, functional characterization of genes in pepper has increasingly relied on heterologous genetic transformation systems, including model organisms such as *A. thaliana* [41] or closely related crop species such as tomato [42]. Thus, in this study, the identification of the gene function of *CaPEX3* in pepper was achieved through VIGS, which is a mature method of transient transformation applied in pepper [42]. The expression level of the target gene can be measured using qPCR, which is normalized with reference genes. Multiple reference genes can yield more accurate results, as the unstable expression of a single reference gene may introduce errors in quantifying gene expression [43]. However, a single reference gene may still be used if it has been proven to exhibit a stable expression in the test tissues [42]. In this study, the expression of *CaPEX3* was detected using well-validated reference genes, and its function was further analyzed. As shown in the results, the silenced expression of *CaPEX3* via VIGS resulted in reduced pollen viability, while the heterologous overexpression of *CaPEX3* enhanced pollen viability, indicating the function of *CaPEX3* in enhancing the vitality of pepper pollen. Furthermore, the exploration of its genetic mechanisms can better reveal *CaPEX3*′s functions.

TFs, as upstream regulatory factors, govern gene expression pathways, and are involved in multiple processes of plant growth and development [44]. Thus, a Y1H screen was performed, and two TFs, *CaTTG1* and *CaNFYC9* were screened out and identified as the upstream regulatory transcription factors of *CaPEX3*. In *A. thaliana*, *AtTTG1*, a WD40 protein, was an essential regulator of late structural genes in flavonoid biosynthesis, and in an *AtTTG1* loss-of-function mutant, seedlings did not accumulate anthocyanidin [45]. The homologous genes of *AtTTG1* were also found to regulate the flavonoid biosynthetic pathway in many plants such as *Malus domestica*, *Arabis alpina*, *Raphanus sativus*, and *O. sativa* [46,47,48,49]. Moreover, NF-YC was able to interact with NF-YB/ NF-YA proteins to bind the *chalcone synthase 1* promoter to further regulate flavonoid biosynthesis in tomato [50]. As reported, flavonoids might maintain intermediate levels of ROS by inhibiting their formation or scavenging ROS [51], playing an important role in pollen germination [16]. Moreover, the flavonoids were sequentially synthesized in both the tapetum and microspores during pollen ontogeny in *A. thaliana* [17]. Furthermore, flavonols improved tomato pollen thermotolerance during germination and tube elongation by maintaining reactive oxygen species homeostasis [14]. Likewise, favonoid biosynthesis played an important role in regulating the pollen fertility of pepper [18]. Therefore, these transcription factors may have effects on pollen development through the regulation of flavonoid biosynthesis and ROS homeostasis. Interestingly, in pepper, CaWD40-91 was able to interact with CaAN1 and CaDYT1, an upstream transcription factor that regulated the development of anthers, to form a novel complex CaAN1-CaDYT1-CaWD40-91, playing roles in anthocyanin biosynthesis and genic male sterility [52]. Taken together, these results indicate that the subject of whether *CaPEX3* is included in the cascading network regulated by DYTI for pepper pollen development is worthy of further study.

In addition, *TTG1* and *NFYC9* were also involved in the regulation of responses to abiotic stresses. In *Setaria italica*, ectopic expression of *SiTTG1* completely restored the salt and sucrose sensitivity phenotypes observed in the *ttg1* mutants, indicating its function in regulating plant response to these stresses [53]. Likewise, after the heterologous expression of *Mangifera indica MiTTG1* in *A. thaliana*, the plants were more adapted to abiotic stresses (mannitol, salt and drought stress) in terms of promoted root hairs and root lengths [54]. In addition, in *A. thaliana*, overexpression of *NF-YC9* conferred salt and osmotic hypersensitivity in early seedling growth [55]. In *Populus tomentosa*, salt-responsive myb transcription factor (SRMT) was able to combine NFYC9 and responsive to desiccation 26 to regulate ABA-dependent salt stress response signaling [56]. Furthermore, a HIPP26-like-NFYC9-SRMT module was also revealed to play roles in regulating drought response [57]. As reported, abiotic stresses, such as heat, cold, salt, and drought, can induce oxidative stress, which can trigger excessive accumulation of ROS and cause serious damage to cells, including protein denaturation, lipid peroxidation, DNA mutations, and even programmed cell death [58,59]. In most plants, meiosis to microspore development seems to be the process that is most sensitive to environmental stress conditions, which affect the quantity and morphology of pollen, cell wall structure, and pollen metabolism, leading to pollen abortion and male sterility [58,60]. Notably, *LRX*s were reported to play important roles in resisting stresses [61]. Thus, under environmental stress, *CaPEX3* may be involved in the regulatory pathways of these transcription factors to maintain ROS homeostasis, enhance resistance, and preserve the normal growth and vitality of pollen.

In summary, it was hypothesized that *CaPEX3* may function as a pivotal hub, integrating diverse signals originating from both intracellular (e.g., flavonoids) and extracellular (e.g., stresses) sources and thereby precisely modulating pollen development and function under complex stress conditions. Certainly, these potential functional mechanisms require further experimental validation, which constitutes a key focus of our future research endeavors. Furthermore, after clarifying that *CaPEX3* and these transcription factors can enhance pollen viability, the overexpressed transgenic materials could be used to develop pepper lines with high pollen vitality, thereby further improving hybridization efficiency or establishing a haploid breeding system to lay the foundation for creating new varieties. Additionally, gene editing or silencing techniques could be employed to attempt the creation of male sterile materials, providing ideal resources for pepper hybrid breeding and gene mapping studies.

## 4. Materials and Methods

### 4.1. Plant Materials and Growth Conditions

Pepper (*C. annuum*) cv. Jinjiao 204 and tomato (*S. lycopersicum*) cv. Micro-Tom seeds were germinated at 28 °C in a light incubator. After five days, seedlings were transplanted into pots filled with a 2:1:1 mixture of soil, vermiculite, and perlite, and cultivated in a growth chamber (16 h^−1^ light/8 h^−1^ dark, 23/20 °C day/night, 150 µmol·m^−2^·s^−1^). Four-leaf pepper seedlings were used for the VIGS assay [42], and Micro-Tom seedlings were used for genetic transformation through the leaf disk method described in Ref. [62]. The leaves and flowers of eight-week-old pepper were collected, directly frozen in liquid nitrogen, and stored at −80 °C until DNA and RNA extraction for cloning. *Nicotiana benthamiana* seeds were germinated in soil and grown in the above growth chamber. Six-week-old *N. benthamiana* plants were used for Dual-LUC assays.

### 4.2. DNA and RNA Extraction

The genomic DNA of pepper flowers was isolated with the CTAB plant genome DNA rapid extraction kit (Aidlab Biotech, Beijing, China) according to the manufacturer’s protocol. The total RNA of pepper leaves and flowers were extracted using the RNAiso Plus reagent kit (TaKaRa, Shiga, Japan) according to the manufacturer’s instruction. The quality and integrity of RNA were detected by observing whether there were intact 28 s and 18 s rRNA bands through 2% agarose gel electrophoresis [63].

### 4.3. First-Strand cDNA Synthesis, PCR Amplification and Product Elution, Recombinant Plasmid Construction and Sequencing

First-strand cDNA synthesis was performed on 2 μg of total RNA using the M-MLV reverse transcriptase kit (Transgene, Beijing, China). The 2000 bp promoter sequence of *CaPEX3* upstream of the translation initiation site (ATG) was amplified from the pepper DNA by PCR with 2X Xerox PCR Master Mix (Biomed, Beijing, China). Using pepper cDNA as a template, the amplifications of gene fragments were performed by PCR with the above PCR mix. All the primers used are designed by Primer Premier 5.0, and listed in Table S1. The PCR volume contained 12.5 µL of 2X Xerox PCR Master Mix, 2 µL of template, 1 µL of forward primer (10 µM), 1 µL of reverse primer (10 µM), and 8.5 µL of ddH_2_O; the total volume was 25 µL. The cycling conditions were as follows: initial denaturation at 98 °C for 1 min; 38 cycles of denaturation at 98 °C for 5 s, annealing at 58 °C for 20 s, and extension at 72 °C for 15 s; a final extension at 72 °C for 5 min; thereafter, the mixture was maintained at 4 °C. The size of PCR product was detected by 1.5% agarose gel electrophoresis, and the correct band was purified using the TIANquick Midi Purification Kit (TIANGEN Biotech. Co., Ltd., Beijing, China) and ligased into the pEASY^®^-T1 simple cloning vector (TransGen, Beijing, China) according to the manufacturer’s instructions. Then, the recombinant vector was transformed into *Escherichia coli* DH5α competent cells (Zoman Biotechnology, Beijing, China) by the method of heat-shock. The mixture (DH5α competent cells and recombinant vector) was incubated on ice for 30 min, followed by a 42 °C water bath for 90 s and an ice bath for 2 min. An amount of 500 µL of antibiotic-free LB liquid medium was added to the sample, and it was incubated at 37 °C with shaking at 180 rpm for 1 h. The culture was plated onto LB solid medium containing the selective antibiotic and cultured overnight at 37 °C. Several resistant colonies were identified by PCR and the PCR-positive colonies were used to perform the isolation of plasmid DNA with the TIANprep Mini Plasmid Kit (TIANGEN Biotech. Co., Ltd., Beijing, China), and the DNA was sequenced by the Sangon Biotechnology Co., Ltd. (Shanghai, China).

### 4.4. Quantitative PCR (qPCR)

qPCR was carried out using TB Green^®^ Premix Ex Taq™ II (TaKaRa, Shiga, Japan) on QuantStudio 3 (Applied Biosystems, USA). The qPCR volume contained 12.5 µL of TB Green Premix Ex Taq II FAST qPCR (2X), 2 µL of cDNA, 1 µL of forward primer (10 µM), 1 µL of reverse primer (10 µM), and 8.5 μL of ddH_2_O; the total volume was 25 µL. The cycling conditions of qPCR were 95 °C for 30 s, followed by 45 cycles of 15 s at 95 °C, 30 s at 60 °C, and 30 s at 72 °C. *CaUBI3* (GenBank accession number, AY486137.1) [64] and *SlActin* (Solyc11g005330) [42], which were detected to be stable in expression under the test plants, were used as the internal reference genes for pepper and tomato, respectively, and the primers used are shown in Table S1. The relative expression level of genes was calculated by the 2^−△△Ct^ method. Three biological replicates, each with three technical replicates, were analyzed, and the results are presented as means ± SD. Statistical analysis was performed with SPSS 21.0 using analysis of variance.

### 4.5. GUS Fusion Vector Construction and Plant Transformation

The 2000 bp promoter sequence of *CaPEX3* upstream of ATG was amplified with pepper DNA and cloned into the plant expression vector pCAMBIA-1300 (Pujie Biology, Shanghai, China) using Xba I and BamH I restriction sites to generate *CaPEX3* promoter::*GUS* via ClonExpress^®^ II One Step Cloning Kit (Vazyme Biotech Co., Ltd., Nanjing, China) with the primers (Table S1). The recombinant vector was identified by the above-mentioned PCR method and sequenced. Then, the mixture (*Agrobacterium tumefaciens* strain *GV3101* competent cells (Zoman Biotechnology, Beijing, China) and 4 μL of plasmid DNA) was incubated on ice for 5 min, followed by a 5 min exposure to liquid nitrogen. Subsequently, the sample was subjected to a 37 °C water bath for 5 min, and then it was returned it to ice for an additional 5 min. An amount of 700 μL of antibiotic-free LB liquid medium was added to the sample, and it was incubated at 28 °C with shaking at 200 rpm for 2–3 h. The culture was plated onto LB solid medium containing the selective antibiotic evenly at 28 °C for 48–72 h. Selecting a single colony for identification proved that the recombinant plasmid had been transferred into *GV3101*. *Agrobacterium*-mediated tomato transformation was performed via leaf dish transformation using cotyledons of Micro-Tom as explants according to the method described before [62]. The WT plants were used as a negative control, and the positive transgenic plants were determined by PCR and qPCR for subsequent phenotypic analysis.

### 4.6. GUS Histochemical Staining

The expression of *GUS* was analyzed using a GUS staining kit (SL7160, Coolaber, Beijing, China) according to the manufacturer’s instruction. Flower-related tissue samples from WT and transgenic tomato lines of *CaPEX3 promoter*::*GUS* (T1-T3) were fully immersed in the GUS staining solution (1mg·mL^−1^ 5-bromo-4-chloro-3-indolyl-glucronide, X-Gluc, pH 7.0) and incubated overnight at 37 °C. Subsequently, the samples were transferred to 70% ethanol for decolorization; this was repeated 2 to 3 times until the negative control tissues became visibly white. The staining patterns were then examined under a microscope (Nikon DS-Ri2, Tokyo, Japan). Six plants were observed for WT and each transgenic line, respectively, and three biological replicates were conducted.

### 4.7. VIGS Assay

pTRV1 and pTRV2 vectors (Towin biotechnology, Wuhan, Beijing) were used for VIGS assay [42]. The full length of the coding sequence of *CaPEX3* (1581 bp) is available on NCBI (GenBank accession number, PV577547). A 300 bp fragment of *CaPEX3* (corresponding to positions +66 to +365 in the *CaPEX3* coding region) was selected by the SNG VIGS TOOL (https://vigs.solgenomics.net/, accessed on 20 November 2021) and amplified from the pepper cDNA by PCR using BamH I and Sac I restriction sites to generate pTRV2:*CaPEX3* with the primers (Table S1). Then, the recombinant vector, pTRV1, and pTRV2 were transformed into the *GV3101*, respectively, as mentioned above. The VIGS assay in pepper was performed as described in the previous report [42].

The *Agrobacterium*-resistant colonies containing the above vectors were added to 1 mL of LB liquid medium (50 mg·L^−1^ kanamycin), separately, and shaken at 28 °C and 160 rpm for 12 h. Then, they were transferred to 50 mL of LB liquid medium (50 mg·L^−1^ kanamycin) and shaken overnight at 28 °C and 160 rpm. The cultured bacterial liquids were centrifuged at 5000 rpm for 10 min, and then the bacteria were collected and re-suspended with the MMA buffer (10 mM of 2-Morpholinoethanesulfonic Acid, 10 mM of MgCl_2_, and 150 µM of acetosyringone) as the infection liquid, with the appropriate value of OD600 (0.8). After shaking at 28 °C and 160 rpm for 2 h, the *Agrobacterium* mixtures of pTRV1 and pTRV2 or pTRV2:*CaPEX3* (1:1) were infiltrated into the lower leaves of four-leaf stage plants using a 1 mL needleless syringe. After being cultured for three days in dark conditions, the plants were transferred to light for normal growth. The second inoculation of leaves was carried out when the flower buds appeared following the above method. The uninoculated plants and those infected with pTRV2 were used as control and negative group. The expression level of *CaPEX3* in the flowers at 60 days after inoculation were detected to assess the silencing efficiency of *CaPEX3*, and the pollens from three plants of the control, negative group, and *CaPEX3*-silenced plants, respectively, were collected for viability analysis. The experiment was repeated three times.

### 4.8. Overexpression Vector Construction and Plant Transformation

The full coding sequence of *CaPEX3* (1581 bp) was amplified from pepper cDNA and cloned into the plant expression vector PHG (PuJie Biology, Shanghai, China) using BamH I and Pst I restriction sites to generate 35S:: *CaPEX3* via a ClonExpress^®^ II One Step Cloning Kit (Vazyme Biotech Co., Ltd., Nanjing, China) with the primers (Table S1). The vector was transformed into the *A. tumefaciens* strain *GV3101* and then transformed into Micro-Tom as described above [62]. The WT plants were used as a negative control, and the positive transgenic plants were determined by PCR and qPCR. After culturing for 70 days, the pollens of the WT and *CaPEX3*-overexpressing plants were collected for analysis.

### 4.9. Pollen Viability Assays

To test in vitro pollen germination, the pollen grains of the pepper and tomato plants used in the VIGS (control, pTRV2, and pTRV2:*CaPEX3*) and overexpression (WT and three transgenic lines: T1, T3, and T6) assays were collected and cultured under dark room conditions at 25 °C. The culture medium for pepper was 5% sucrose + 100 mg·L^−1^ boric acid +5 mg·L^−1^ GA3 +100 mg·L^−1^ calcium nitrate+ 0.8% agar. The culture medium for tomato was 120 g·L^−1^ sucrose + 120 mg·L^−1^ boric acid + 4 mg·L^−1^ GA3 + 0.5 mg·L^−1^ thiamine + 1% agar. The pollen grains were counted after 3 h of incubation. Pollen viability was determined using 1% 2, 3, 5-triphenyltetrazolium chloride (TTC) solution (Coolaber, Beijing, China) [65]. After being placed at 35 °C for 15 min, the pollen staining situation was observed. Red pollen grains indicate vigorous pollen; the darker the color, the stronger the vitality [66]. For each treatment, pollens were collected from fifteen flowers from three plants of each line, ten distinct fields of view were observed under an optical microscope (Nikon DS-Ri2, Japan), and the number of pollen grains counted in each field of view was at least 50. Pollen germination rate = number of germinated grains of pollens/total number of pollen grains × 100%. Pollen viability (%) = number of stained pollen grains/total number of observed pollen grains × 100%. Three biological replicates were performed, and statistical analysis was performed with SPSS 21.0 using analysis of variance.

### 4.10. Construction of cDNA Library

The cDNA library was constructed by mixed RNA from the stems, leaves, and flowers of eight-week-old pepper using the method reported before [67]. An amount of 400–500 µg of total RNA was used to isolate mRNA with Fasttrack MAG beads (Thermo Fisher Scientific, MA, USA). The primary library was synthesized using attB2 as a linker. BP Clonase II Mixp (Thermo Fisher Scientific, MA, USA) was used to recombine pDONR222 (ZYbscience, Shanghai, China) with cDNA, which was then electro-transformed into *Escherichia coli* DH10B (Zoman Biotechnology, Beijing, China). The clone number of the primary library was 1.08 × 10^7^ cfu. Then, the plasmid of the primary library was extracted by A PureLink^R^ HiPure Plasmid Filter Midiprep Kit (Thermo Fisher Scientific, MA, USA) and transferred into DH10B to obtain the secondary library. The clone number of the secondary library was 1.04 × 10^7^ cfu, which reached the standards for yeast hybrid library construction.

### 4.11. Y1H Screening and Identification

The 2000 bp promoter sequence of *CaPEX3* upstream of ATG was amplified and cloned into bait vector pHIS2 (Takara, Shiga, Japan) with the primers (Table S1), generating *CaPEX3pro*-pHIS2. The prey library was constructed by ligating the cDNA library into the pGADT7 vector. For the Y1H assay, the bait and prey vectors were then co-introduced into the yeast strain Y187 (Zoman Biotechnology, Beijing, China), and the screening was conducted as described previously [67]. The positive clones were sequenced, and the transcription factors were predicted by PlantRegMap/PlantTFDB v5.0 (https://planttfdb.gao-lab.org/prediction.php, accessed on 8 December 2022).

Then, the transcription factors *CaTTG1* and *CaNFYC9* were screened out, and their relationships with *CaPEX3* promoter were verified by Y1H point-to-point assays. The full coding sequences of *CaTTG1/CaNFYC9* were amplified and cloned into the pGADT7 vector with primers (Table S1). Then, the *CaTTG1/CaNFYC9*-pGADT7 and *CaPEX3pro*-pHIS2 vectors were separately co-transformed into Y187, and the yeast cells were plated on SD/-Leu/-Trp media and incubated for 3–5 days. The positive clones were selected and transferred to SD/-Leu-Trp-His media with 120 and 150 mM of 3-amino-1, 2, 4-triazole (3-AT), separately, followed by another 3–5 days of incubation. Rec2-53-pGADT7 (Takara, Shiga, Japan) and p53-pHIS2 (Takara, Shiga, Japan) were co-transformed into Y187 as positive control. pGADT7 and p53-pHIS2 (Takara, Shiga, Japan) were co-transformed into Y187 as negative control. Interaction was proven based on the growth of the yeast colony.

### 4.12. Dual-LUC Assays

The full coding sequences of *CaTTG1/CaNFYC9* were individually inserted into the pGreenII 62-SK vector (Miaoling biology, Wuhan, China) to generate the effector constructs (*CaTTG1/CaNFYC9*-pGreenII 62-SK). The 2000 bp promoter sequence of *CaPEX3* upstream of ATG was cloned into pGreenII 0800-LUC vectors (Miaoling biology, Wuhan, China) to generate the reporter constructs (*CaPEX3pro*-pGreenII 0800-LUC). The above effector and reporter constructs were individually transformed into *GV3101*. pGreenII 62-SK and *CaPEX3pro*-pGreenII 0800-LUC were used as negative control. *CaTTG1/CaNFYC9*-pGreenII 62-SK and *CaPEX3pro*-pGreenII 0800-LUC were used as the experimental group. *N. benthamiana* leaves were infected with the mixed *Agrobacterium* strains of the negative control and experimental group, respectively. After a 72 h incubation at 23 °C, fluorescence was detected by Dual-luciferase Reporter Assay System (Promega, Madison, WI, USA). Promoter activity was represented by the LUC/REN ratio following normalization [67]. For each combination, LUC/REN ratios from at least three independent transformations were determined. All the primers used are listed in Table S1.

## 5. Conclusions

In this study, GUS histochemical staining confirmed that *CaPEX3* was expressed in petals, sepals, anthers, and pollen. Furthermore, VIGS for *CaPEX3* in pepper resulted in significantly reduced pollen viability, while the overexpression of *CaPEX3* in tomato enhanced pollen viability, revealing its function in regulating pollen vitality. In addition, the identified upstream regulatory transcription factors of *CaPEX3* helped explain the mechanism underlying the regulation of pollen viability, which provided genetic resources and a theoretical basis for the research on the regulation of pollen viability in pepper.

## Data Availability

The original contributions presented in this study are included in the article/Supplementary Material. Further inquiries can be directed to the corresponding author.

## Supplementary Materials

The following supporting information can be downloaded at: https://www.mdpi.com/article/10.3390/plants14223441/s1, Table S1: Primers used in this study.

## Abbreviations

The following abbreviations are used in this manuscript:

PEX	Pollen extensin-like
LRX	Leucine-rich repeat extensin
VIGS	Virus-induced gene silencing
TTG1	Transparent testa glabra 1
NFYC9	Nuclear transcription factor Y subunit C-9
Y1H	Yeast one-hybrid
Dual-LUC	Dual-luciferase reporter
ROS	Reactive oxygen species
TF	Transcription factor
DYT1	Dysfunctional tapetum 1
TDF1	Tapetal development and function 1
AMS	Aborted microspores
MS	Male sterile
RALF	Rapid alkalinization factor
WT	Wild type
TTC	2, 3, 5-triphenyltetrazolium chloride
SRMT	Salt responsive myb transcription factor
3-AT	3-amino-1, 2, 4-triazole

## References

[1] Dobón–Suárez A., Zapata P.J., García-Pastor M.E. (2025). A comprehensive review on characterization of pepper seeds: Unveiling potential value and sustainable agrifood applications. Foods.

[2] Du K., Zhang D., Ma J., Dan Z., Wen X., Lv W., Yang L., Bao L., Li Y., Chen G. (2025). Effect of ultra-low temperature storage on the viability of pepper pollen and its implications for hybrid breeding. Front. Plant Sci..

[3] Wang Z.Y., Zhang C.P. (2025). An improved chilli pepper flower detection approach based on YOLOv8. Plant Methods.

[4] Meng D., Liu C., Chen S., Jin W. (2021). Haploid induction and its application in maize breeding. Mol. Breed..

[5] Lantos C., Jancsó M., Székely Á., Nagy É., Szalóki T., Pauk J. (2022). Improvement of anther culture to integrate doubled haploid technology in temperate rice (*Oryza sativa* L.) breeding. Plants.

[6] Shmykova N., Domblides E., Vjurtts T., Domblides A. (2020). Haploid embryogenesis in isolated microspore culture of carrots (*Daucus carota* L.). Life.

[7] Mestinšek Mubi Š., Kunej U., Vogrinčič V., Jakše J., Murovec J. (2024). The effect of phytosulfokine alpha on haploid embryogenesis and gene expression of *Brassica napus* microspore cultures. Front. Plant Sci..

[8] Galán-Ávila A., García-Fortea E., Prohens J., Herraiz F.J. (2021). Microgametophyte development in *Cannabis sativa* L. and first androgenesis induction through microspore embryogenesis. Front. Plant Sci..

[9] Jaingulueam T., Suwor P., Saetiew K., Tsai W.S., Techawongstien S., Tarinta T., Kumar S., Jeeartid N., Chatchawankanphanich O., Kramchote S. (2023). Characterization of floral bud and anther development with stages of microspore in chili pepper (*Capsicum annuum*). Acta Hortic..

[10] Fernando D.D., Lazzaro M.D., Owens J.N. (2005). Growth and development of conifer pollen tubes. Sex. Plant Reprod..

[11] Gómez J.F., Talle B., Wilson Z.A. (2015). Anther and pollen development: A conserved developmental pathway. J. Integr. Plant Biol..

[12] Shin J.M., Yuan L., Ohme-Takagi M., Kawashima T. (2021). Cellular dynamics of double fertilization and early embryogenesis in flowering plants. J. Exp. Zool. Part B Mol. Dev. Evol..

[13] Müller F., Rieu I. (2016). Acclimation to high temperature during pollen development. Plant Reprod..

[14] Postiglione A.E., Delange A.M., Ali M.F., Wang E.Y., Houben M., Hahn S.L., Khoury M.G., Roark C.M., Davis M., Reid R.W. (2024). Flavonols improve tomato pollen thermotolerance during germination and tube elongation by maintaining reactive oxygen species homeostasis. Plant Cell.

[15] Mehmood M., Tanveer N.A., Joyia F.A., Ullah I., Mohamed H.I. (2025). Effect of high temperature on pollen grains and yield in economically important crops: A review. Planta.

[16] Lan X., Yang J., Abhinandan K., Nie Y., Li X., Li Y., Samuel M.A. (2017). Flavonoids and ROS play opposing roles in mediating pollination in ornamental kale (*Brassica oleracea* var. *acephala*). Mol. Plant.

[17] Xue J.S., Qiu S., Jia X.L., Shen S.Y., Shen C.W., Wang S., Xu P., Tong Q., Lou Y.X., Yang N.Y. (2023). Stepwise changes in flavonoids in spores/pollen contributed to terrestrial adaptation of plants. Plant Physiol..

[18] Zhang Z., Liu Y., Yuan Q., Xiong C., Xu H., Hu B., Suo H., Yang S., Hou X., Yuan F. (2022). The bHLH1-DTX35/DFR module regulates pollen fertility by promoting flavonoid biosynthesis in *Capsicum annuum* L.. Hortic. Res..

[19] Phan H.A., Li S.F., Parish R.W. (2012). MYB80, a regulator of tapetal and pollen development, is functionally conserved in crops. Plant Mol. Biol..

[20] Gu J.N., Zhu J., Yu Y., Teng X.D., Lou Y., Xu X.F., Liu J.L., Yang Z.N. (2014). DYT1 directly regulates the expression of TDF1 for tapetum development and pollen wall formation in *Arabidopsis*. Plant J..

[21] Gawande N.D. (2025). MYB80 and TEK: Dynamic duo regulating callose wall degradation and pollen exine development. Plant Physiol..

[22] Li D.D., Xue J.S., Zhu J., Yang Z.N. (2017). Gene regulatory network for tapetum development in *Arabidopsis thaliana*. Front. Plant Sci..

[23] Wei C., Zhang R., Yue Z., Yan X., Cheng D., Li J., Li H., Zhang Y., Ma J., Yang J. (2021). The impaired biosynthetic networks in defective tapetum lead to male sterility in watermelon. J. Proteom..

[24] Baumberger N., Doesseger B., Guyot R., Diet A., Parsons R.L., Clark M.A., Simmons M.P., Bedinger P., Goff S.A., Ringli C. (2003). Whole–genome comparison of leucine-rich repeat extensins in *Arabidopsis* and rice. A conserved family of cell wall proteins form a vegetative and a reproductive clade. Plant Physiol..

[25] Zhao C., Zayed O., Yu Z., Jiang W., Zhu P., Hsu C.C., Zhang L., Tao W.A., Lozano-Durán R., Zhu J.K. (2018). Leucine-rich repeat extensin proteins regulate plant salt tolerance in *Arabidopsis*. Proc. Natl. Acad. Sci. USA.

[26] Zhou J., Rumeau D., Showalter A.M. (1992). Isolation and characterization of two wound-regulated tomato extensin genes. Plant Mol. Biol..

[27] Baumberger N., Ringli C., Keller B. (2001). The chimeric leucine-rich repeat/extensin cell wall protein LRX1 is required for root hair morphogenesis in *Arabidopsis thaliana*. Genes Dev..

[28] Wang X., Wang K., Yin G., Liu X., Liu M., Cao N., Duan Y., Gao H., Wang W., Ge W. (2018). Pollen-expressed leucine-rich repeat extensins are essential for pollen germination and growth. Plant Physiol..

[29] Sede A.R., Borassi C., Wengier D.L., Mecchia M.A., Estevez J.M., Muschietti J.P. (2018). *Arabidopsis* pollen extensins LRX are required for cell wall integrity during pollen tube growth. FEBS Lett..

[30] Fabrice T.N., Vogler H., Draeger C., Munglani G., Gupta S., Herger A.G., Knox P., Grossniklaus U., Ringli C. (2018). LRX proteins play a crucial role in pollen grain and Pollen tube cell wall development. Plant Physiol..

[31] Dai H., Li Y., Liu S., Lin L., Wu J., Zhang Z., Peng Q., Li N., Zhang X. (2021). Effect of extensin-like OsPEX1 on pollen fertility in rice. Hereditas.

[32] Mecchia M.A., Santos-Fernandez G., Duss N.N., Somoza S.C., Boisson-Dernier A., Gagliardini V., Martínez-Bernardini A., Fabrice T.N., Ringli C., Muschietti J.P. (2017). RALF4/19 peptides interact with LRX proteins to control pollen tube growth in *Arabidopsis*. Science.

[33] Moussu S., Broyart C., Santos-Fernandez G., Augustin S., Wehrle S., Grossniklaus U., Santiago J. (2020). Structural basis for recognition of RALF peptides by LRX proteins during pollen tube growth. Proc. Natl. Acad. Sci. USA.

[34] Moussu S., Lee H.K., Haas K.T., Broyart C., Rathgeb U., De Bellis D., Levasseur T., Schoenaers S., Fernandez G.S., Grossniklaus U. (2023). Plant cell wall patterning and expansion mediated by protein–peptide–polysaccharide interaction. Science.

[35] Dünser K., Gupta S., Herger A., Feraru M.I., Ringli C., Kleine-Vehn J. (2019). Extracellular matrix sensing by FERONIA and leucine-rich repeat extensins controls vacuolar expansion during cellular elongation in *Arabidopsis thaliana*. EMBO J..

[36] Cheung A.Y. (2024). FERONIA: A receptor kinase at the core of a global signaling network. Annu. Rev. Plant Biol..

[37] Wu L., Liu X., Zhang M.Y., Qi K.J., Jiang X.T., Yao J.L., Zhang S.L., Gu C. (2023). Self S-RNase inhibits ABF-LRX signaling to arrest pollen tube growth to achieve self-incompatibility in pear. Plant J..

[38] Zang Q.L., Liu L., Liu H., Wang J., Wang M., Cheng Y. (2024). Cloning and expression analysis of *CaPEX3* in pepper. Acta Bot. Boreali–Occident. Sin..

[39] Rubinstein A.L., Broadwater A.H., Lowrey K.B., Bedinger P.A. (1995). Pex1, a pollen-specific gene with an extensin-like domain. Proc. Natl. Acad. Sci. USA.

[40] Tian H., Fan G., Xiong X., Wang H., Zhang S., Geng G. (2024). Characterization and transformation of the *CabHLH18* gene from hot pepper to enhance waterlogging tolerance. Front. Plant Sci..

[41] Xiao J., Wang D., Liang L., Xie M., Tang Y., Lai Y.S., Sun B., Huang Z., Zheng Y., Li H. (2024). CaMYB80 enhances the cold tolerance of pepper by directly targeting *CaPOA1*. Hortic. Res..

[42] Mao L., Shen Y., Cui Q., Huang Y., Zhang X., Lv J., Xing W., Zhang D., Fang N., Chen D. (2025). The IQ67-domain protein IQD1 regulates fruit shape through complex multiprotein interactions in pepper (*Capsicum annuum* L.). Plant Biotechnol. J..

[43] Hu X., Zhou C., Pan J., Wu W., Wu S., Yan X., Wang C., Zhu Q. (2025). Screening and Validation of Stable Reference Genes for Real-Time Quantitative PCR in *Indocalamus tessellatus* (Munro) P. C. Keng Under Multiple Tissues and Abiotic Stresses. Forests.

[44] Strader L., Weijers D., Wagner D. (2022). Plant transcription factors—Being in the right place with the right company. Curr. Opin. Plant Biol..

[45] Zhang B., Schrader A. (2017). TRANSPARENT TESTA GLABRA 1-dependent regulation of flavonoid biosynthesis. Plants.

[46] Brueggemann J., Weisshaar B., Sagasser M. (2010). A WD40-repeat gene from *Malus* x *domestica* is a functional homologue of *Arabidopsis thaliana* TRANSPARENT TESTA GLABRA1. Plant Cell Rep..

[47] Chopra D., Wolff H., Span J., Schellmann S., Coupland G., Albani M.C., Schrader A., Hulskamp M. (2014). Analysis of *TTG1* function in *Arabis alpina*. BMC Plant Biol..

[48] Lim S.H., Kim D.H., Lee J.Y. (2022). RsTTG1, a WD40 Protein, interacts with the bHLH transcription factor RsTT8 to regulate anthocyanin and proanthocyanidin biosynthesis in *Raphanus sativus*. Int. J. Mol. Sci..

[49] Zhu C., Yang X., Chen W., Xia X., Zhan Z., Qing D., Nong B., Li J., Liang S., Luo S. (2024). WD40 protein OsTTG1 promotes anthocyanin accumulation and CBF transcription factor–dependent pathways for rice cold tolerance. Plant Physiol..

[50] Wang J., Li G., Li C., Zhang C., Cui L., Ai G., Wang X., Zheng F., Zhang D., Larkin R.M. (2021). NF-Y plays essential roles in flavonoid biosynthesis by modulating histone modifications in tomato. New Phytol..

[51] Agati G., Azzarello E., Pollastri S., Tattini M. (2012). Flavonoids as antioxidants in plants: Location and functional significance. Plant Sci..

[52] Tang P., Huang J., Wang J., Wang M., Huang Q., Pan L., Liu F. (2024). Genome-wide identification of CaWD40 proteins reveals the involvement of a novel complex (CaAN1-CaDYT1-CaWD40-91) in anthocyanin biosynthesis and genic male sterility in *Capsicum annuum*. BMC Genom..

[53] Liu K., Qi S., Li D., Jin C., Gao C., Duan S., Feng B., Chen M. (2017). TRANSPARENT TESTA GLABRA 1 ubiquitously regulates plant growth and development from *Arabidopsis* to foxtail millet (*Setaria italica*). Plant Sci..

[54] Tan L., Salih H., Htet N.N.W., Azeem F., Zhan R. (2021). Genomic analysis of WD40 protein family in the mango reveals a TTG1 protein enhances root growth and abiotic tolerance in *Arabidopsis*. Sci. Rep..

[55] Bi C., Ma Y., Wang X.F., Zhang D.P. (2017). Overexpression of the transcription factor *NF-YC9* confers abscisic acid hypersensitivity in *Arabidopsis*. Plant Mol. Biol..

[56] Tong S., Wang Y., Chen N., Wang D., Liu B., Wang W., Chen Y., Liu J., Ma T., Jiang Y. (2022). PtoNF–YC9–SRMT–PtoRD26 module regulates the high saline tolerance of a triploid poplar. Genome Biol..

[57] Chen K., Tong S., Huang H., Li J., Shi W., Li Y., Wang W., Xu L., Luo T., Zhao L. (2025). HIPP26L–NF–YC9–SRMT module regulates drought response in poplar. Cell Rep..

[58] Chaturvedi P., Wiese A.J., Ghatak A., Záveská Drábková L., Weckwerth W., Honys D. (2021). Heat stress response mechanisms in pollen development. New Phytol..

[59] Xie D.L., Zheng X.L., Zhou C.Y., Kanwar M.K., Zhou J. (2022). Functions of redox signaling in pollen development and stress response. Antioxidants.

[60] Zinta G., Khan A., AbdElgawad H., Verma V., Srivastava A.K. (2016). Unveiling the redox control of plant reproductive development during abiotic stress. Front. Plant Sci..

[61] Zhao C., Jiang W., Zayed O., Liu X., Tang K., Nie W., Li Y., Xie S., Li Y., Long T. (2020). The LRXs-RALFs-FER module controls plant growth and salt stress responses by modulating multiple plant hormones. Natl. Sci. Rev..

[62] Cao H., Chen J., Yue M., Xu C., Jian W., Liu Y., Song B., Gao Y., Cheng Y., Li Z. (2020). Tomato transcriptional repressor MYB70 directly regulates ethylene–dependent fruit ripening. Plant J..

[63] Arunachalam K., Sreeja P.S. (2025). Chapter 18: Agarose Gel Electrophoresis for RNA Integrity Assessment. Advanced Cell and Molecular Techniques: Protocols for In Vitro.

[64] Zhou L., Yang S., Chen C., Li M., Du Q., Wang J., Yin Y., Xiao H. (2023). *CaCP15* Gene Negatively Regulates Salt and Osmotic Stress Responses in *Capsicum annuum* L.. Genes.

[65] Li W., Yang C., Li J., Huang L., Guo J., Feng F. (2025). Optimization of in vitro germination, viability tests and storage of Daylily (*Hemerocallis* spp.) pollen. Plants.

[66] Hao Q., Xu L., Wang H., Liu Q., Wang K. (2022). Evaluation of pollen viability, stigma receptivity and the cross barrier between tropical and hardy water lily cultivars. Flora.

[67] Li X., Zhang M.S., Zhao L.Q., Ling-Hu Q.Q., Xu G. (2023). The study on interacting factors and functions of *GASA6* in *Jatropha curcas* L.. BMC Plant Biol..

